# Aspects of Health-Related Factors and Nutritional Care Needs by Survival Stage among Female Cancer Patients in South Korea

**DOI:** 10.1371/journal.pone.0163281

**Published:** 2016-10-03

**Authors:** Yoonsun Lee, Hyunsoo Shin, Eunjoo Bae, Hyunjung Lim

**Affiliations:** 1 Department of Medical Nutrition, Graduate School of East-West Medical Science, Kyung Hee University, Yong-In, Gyenggi-do, 17104, Republic of Korea; 2 Research Institute of Medical Nutrition, Kyung Hee University, Seoul, 02447, Republic of Korea; 3 Department of Radiation Oncology, CHA Bundang Medical Center, CHA University, Bundang, Gyenggi-do, 13496, Republic of Korea; 4 Department of Food and Nutrition Service, CHA Bundang Medical Center, CHA University, Bundang, Gyenggi-do, 13496, Republic of Korea; Fu Jen Catholic University, TAIWAN

## Abstract

**Purpose:**

This study examined diet-related problems and needs associated with nutritional care according to survival stage in Korean female cancer survivors.

**Methods:**

186 outpatients (breast or gynecologic cancer survivors) recruited. Subjects were classified as (1) extended stage (ES, 2–5 years from diagnosis) and (2) long-term stage (LS, ≥5 years from diagnosis). Eating habits, changes in health related factors, nutritional needs, and quality of life were investigated.

**Results:**

43% of ES survivors had diet-related problems (p = .031); ES group reported dyspepsia and LS group reported anorexia/nausea as the major problem. Half of ES survivors had taste change, decreasing amount of intake, and reduced quality of life (p < .05). The LS group had a greater preference for sweet tastes than the ES group. According to their diagnosis, ES survivors with breast cancer gained weight (27.1%), whereas ES survivors with gynecologic cancer lost their body weight (34.5%) significantly. LS breast cancer patients showed great food preference for vegetables, whereas those with gynecologic cancer showed an increased preference for fish, meat and grain. Approximately 90% of survivors demanded nutritional care regarding restricted foods, preventing recurrence, particularly in ES survivors (p < .01). Moreover, main factors for nutritional care needs were body weight control for breast cancer and food environment for gynecologic cancer.

**Conclusion:**

Survivors have different aspects of diet-related problems by survival stage as dyspepsia in ES and anorexia in LS. ES stage had changes in dietary patterns and their food consumption have decreased. Most of survivors have demanded nutritional care regardless of survival stage. These features of each stage should be considered to improve their health.

## Introduction

Over 2.4 million South Korean women were diagnosed with a female cancer (site of breast, cervix uteri, corpus uteri, or ovary) in 2012. While the cancer survival rate has increased as a result of early detection of disease and innovative developments of medical technology [1,2]. The 5-year relative cancer survival rates among Korean women increased from 53.4% in 1993–1995 to 76.6% in 2008–2012, with high survival rates for breast cancer (91.1%), cervical cancer (80.3%), and ovarian cancer (61.9%) [2]. The Korea National Cancer Center used the classification of Mullan’s cancer survivorship stages according to the period after initial diagnosis and treatment phase as acute stage, extended stage, and long-term stage [3]. The proportion of Korean cancer patients between 2 and 5 years and more than 5 years after initial diagnosis in 2013 was reported to be 31% and 41%, respectively [2].

In addition, cancer survivors are known to have a low quality of life [4–6] generally and their major concerns are managing side effects, recovery of physical function, and health care to prevent recurrence after medical treatments [7]. Nutritional status and food intakes are meaningful aspects of health promoting behavior and can reduce the risk of recurrence [8]. It is also reported that health behaviors have positive effects for improving symptoms and reducing stress in long-term breast cancer survivors [9]. Especially, a high demand for nutritional care among health promoting behaviors has been reported for breast or gynecologic cancer patients and survivors [10–15]. Consequently, understanding health-related problems of each stage of cancer survivorship is important and might improve survivors’ quality of life [16].

However, previous studies of female cancer survivors mainly focused on their quality of life, health promotion, nutritional status, and physical activities. There are few studies on diet-related changes and nutritional care needs among female cancer survivors.

Therefore, the present study investigated diet-related problems and nutritional care needs according to the survival stage among female cancer survivors in South Korea.

## Materials and Methods

### 1. Design and subjects

#### 1.1 Ethic statement

This study protocol had been approved by Bundang CHA Medical Center, Hospital Ethic Committee (Bundang, Kyeonggi-do, South Korea) (IRB No.IRB#BD-2014-086). All participants signed a written informed consent prior to participate in this study.

#### 1.2 Design and subsects

This cross-sectional study included 212 female cancer survivors aged 18–65 years who attended the outpatient unit of Bundang Cha Medical Center (Bundang, Kyeonggi-do, South Korea) between July and August in 2014. Among them, 26 subjects were excluded because of incomplete responses to the questionnaire or age greater than 65, finally 186 subjects were included in the analysis.

We used the classification of Mullen’s cancer survivorship stages according to the period after initial diagnosis and treatment phase as follows: acute, extended and long-term stage. To compare each stage of cancer survivors’ health related factors and nutritional care needs after medical treatment, we recruited subjects who complete the surgery and medical treatments. Two stages of survivorship were included according to the time elapsed since initial diagnosis: extended stage (between 2–5 years, the end of medical therapies and when the patient shows recovery) and long-term stage (more than 5 years, when the possibility of recurrence is markedly decreased, and almost no cancer cells are active). The patients were clinically diagnosed with breast cancer (diagnosis of breast cancer or cancer in situ of breast) or gynecologic cancer (diagnosis of uterine cancer, endometrial cancer, cancer in situ of uterine, uterine cervical cancer, or ovarian cancer). Initial diagnosis was at least 2 years before initiation of this study, and all medical treatment was completed except for hormonal therapy.

Subjects were divided according to the clinical diagnosis into groups of breast cancer survivors and gynecologic cancer survivors. Each group was further classified into two subgroups according to the period of survival: extended stage (ES) and long-term stage (LS).

### 2. Measures

#### 2.1 General characteristics and medical history

A questionnaire was used to describe the demographics of the study subjects including age, body mass index (BMI, weight [kg]/height [m^2^]; body weight and height was measured before self-report), educational level, monthly income and marital status. The cancer stage at initial diagnosis was reported. Medical history was also assessed with a multiple-choice questionnaire to determine whether the subjects had any other clinically diagnosed diseases.

#### 2.2 Eating habits

To identify usual eating habits over the last 6 months, subjects were asked about the meal frequency per day (with responses ranging from once to more than three times), time regularity of meals in a week (regular; eating regular meals more than 5 days a week, mostly regular; eating regular meals 3–4 days a week, or irregular; eating regular meals less than 2 days a week), eating speed (fast, medium, or slow), frequency of eating out (once a day or more, once or more a week, or less than once a month) and snacking frequency (≥ 3 times a day, twice a day, once a day, or less than once a day). Subjects were also asked whether they have taken functional foods.

#### 2.3 Changes in health related factors

To examine changes in health related factors after surgery and medical treatments, those follow questionnaires are included; dietary problems, changes in the amount of food intake, body weight, dietary pattern, taste and food preference, interests in dietary information, experience of searching for dietary treatments. Each subject was asked whether they have any dietary problems and if so, a subsequent multiple-choice question about the nature of the problem was added. A question about changes in the amount of food intake and the body weight were asked with responses of increased, decreased, or maintained. The women also responded whether they had changes in dietary pattern, taste, or food preference, an interest in dietary information, and experience of searching for dietary treatments with response of “yes” or “no”. In addition, questions about changes in the kinds of food preference (with responses of grain, fruit, vegetable, meat, fish, or nuts) and the kinds of taste preference (with responses of salty, sweet, sour, spicy, or bitter), were included. Six food groups are constituted by Korea Dietary Reference Intakes (KDRIs). Data of changes in the kinds of food and taste preference were collected by multiple-choice questionnaire and then converted into a percentage (%). We used diet-related problems and changes in cancer survivors from a guideline of national cancer information center in Korea and related researches. Then we synthesized those each diet-related problems and changes, and then constitute categories of the survey.

#### 2.4 Nutritional care needs

Nutritional care needs were assessed by questions concerning survivors’ detailed necessity of nutrition care with the response of “yes” or “no”. We synthesize and categorize results of nutrition guidelines of Korean Breast Cancer Society and previous studies related in nutritional problem, needs and guideline for cancer survivors to compose values of this survey. Important factors were investigated with questions “whether those 13 categories are necessity”; amount of food consumed, restricted foods, recipes of healthy foods, general meals, detailed dietary plan, functional foods, body weight, nutritional supplementary drinks, the way to preventing recurrence, nutritional status, purchasing functional foods, food environment, and nutritional counseling. Results were calculated to the percentage, and we describe only the response of “yes” as a figure.

#### 2.5 Quality of life

Quality of life score of the subjects was assessed by the 36-Item Short Form Health Survey (SF-36), which was prepared by Ware & Sherboun and is a proven self-reported scale [17]. The survey had two categories and eight subcategories: physical component summary (physical functioning, role physical, bodily pain, and general health) and mental component summary (vitality, social functioning, role emotional and mental health). The scores for each category, ranging from 0 to 100, were summed. The total was transformed by a standardization grade (mean 50, SD 10) and calculated as an average score. A higher number indicated a better quality of life.

### 3. Statistical analysis

All statistical analyses were performed using the Statistical Package for the Social Sciences (SPSS, Inc., Chicago, IL, USA) version 21.0. Results were expressed as mean and standard deviation (SD) for continuous variables and as number and percentages for discrete variables and multiple-choice question analysis. To compare the means of continuous variables, total, breast, and gynecologic cancer survivors were each classified into extended stage versus long-term stage and compared by t-test. Chi-square test (X^2^-test) was used to verify the significance of discontinuous frequencies. Statistical significance was defined as p < .05. The reliability of the survey was Cronbach's α = 0.719, calculated by SPSS version 21.0.

## Results

### 1. General characteristics and medical history

Mean age of the subjects was 50.3±7.0 years, and the LS group was significantly older than the ES group (p < .05), and the BMI was normal range in all groups. Although most breast cancer survivors were clinically diagnosed in the incipient stage, gynecologic cancer showed a wider range of stage at diagnosis. Overall, the LS group had a greater incidence of other diagnosed disease such as diabetes mellitus (DM), hypertension (HTN) and cardiovascular disease (CVD), compared with the ES group. However, general characteristics and medical history showed no significant differences between ES and LS groups (Table 1.).

**Table 1:** General characteristics according to survival stage among Korean female cancer patients.

Variables	Both			Breast cancer			Gynecologic cancer		
	Extended stage^a^ (n = 120)	Long-term stage (n = 66)	*p*-value	Extended stage (n = 59)	Long-term stage (n = 45)	*p*-value	Extended stage (n = 61)	Long-term stage (n = 21)	*p*-value
Mean age (yrs)	49.2±7.3^b^	52.2±6.1	.003^††^^c^	48.8±6.5	52.7±5.4	.001^††^	49.7±8.0	51.2±7.3	.398
BMI^d^	23.0±4.1	22.8±3.9	.843	23.2±3.7	22.8±4.3	.666	22.8±4.4	22.9±3.2	.924
Educational level									
• <High school	26(21.7)^e^	18(27.2)	.073	9(15.3)	12(26.6)	.482	17(27.9)	6(28.6)	.532
• High school	55(45.8)	29(44.0)		28(47.5)	22(48.9)		27(44.3)	7(33.3)	
• ≥College/University	39(32.5)	19(28.8)		22(37.2)	11(24.5)		17(27.8)	8(38.1)	
Monthly income (10,000 won)									
• High (<200)	74(62.2)	37(56.9)	.486	32(54.2)	27(60.0)	.557	42(70.0)	10(50.0)	.104
• Low (≥200)	45(37.8)	28(43.1)		27(45.8)	18(40.0)		18(30.0)	10(50.0)	
Marital status									
• Married (living with spouse)	90(75.0)	54(81.8)	.453	40(67.8)	36(80.0)	.499	50(82.0)	18(85.7)	.317
• Married (separated)	9(7.5)	2(3.0)		3(5.1)	2(4.4)		6(9.8)	0(0.0)	
• Divorced/widowed	16(13.3)	9(13.6)		12(20.3)	6(13.3)		4(6.6)	3(14.3)	
• Single	5(4.2)	1(1.5)		4(6.8)	1(2.2)		1(1.6)	0(0.0)	

^a^ Extended cancer survivor = between 2 and 5 years after initial diagnosis; Long-term cancer survivor = more than 5 years after initial diagnosis

^b^ Values are mean ± SD

^c^ Significant difference at †† (p<0.01) was found by t-test for continuous variables and * (p<0.05) was found by Chi-square test for categorical variables.

^d^ BMI: Body mass index = body weight (kg) / height (m^2^)

^e^ Values are n (%)

### 2. Eating habits

Over the last six months, nearly 70% of survivors reported having meals three times a day, 80% of survivors reported having regular meals more than three times a week, and 50% of survivors reported spending 10 to 20 minutes on each meal. Approximately 70% of subjects reported snacking more than once a day, and mainly they choose fruits and vegetables for their snacks (data not shown). Among breast cancer survivors, significantly more LS than ES subjects reported taking functional foods (Table 2) (p < .05). The major reason for taking functional foods was recovering health in the ES group and preventing recurrence and improving nutritional status in the LS group. According to the Subjects’ who reported taking functional foods response, they mainly use vitamins (ES = 32.6%, LS = 41.7%), omega-3 (ES = 11.6%, LS = 12.5%) and ginseng products (ES = 9.3%, LS = 18.8%) in sequence (data not shown).

**Table 2:** Changes in health related factors after surgery and medical treatments by the survival stages among Korean female cancer patients.

Variables	Both			Breast cancer			Gynecological cancer		
	Extend stage^a^ (n = 120)	Long-term stage (n = 66)	*p*-value	Extend stage (n = 59)	Long-term stage (n = 45)	*p*-value	Extend stage (n = 61)	Long-term stage (n = 21)	*p*-value
Eating problem^b^^,^^c^	52(43.3)^d^	18(27.3)	.031*^e^	27(45.8)	11(24.4)	.025*	25(41.0)	7(33.3)	.535
Functional foods intake	52(43.3)	36(54.5)	.143	23(39.0)	27(60.0)	.034*	29(47.5)	9(42.9)	.710
Changes in dietary pattern	75(62.5)	42(63.6)	.878	43(72.9)	28(62.2)	.247	32(52.5)	14(66.7)	.258
Changes in taste	55(45.8)	18(27.3)	.013*	32(54.2)	10(22.2)	.001**	23(37.7)	8(38.1)	.975
Changes in food preference	49(40.8)	22(33.3)	.314	30(50.8)	13(28.9)	.024*	19(31.1)	9(42.9)	.329
Interests in dietary information	102(85.0)	56(84.8)	.978	54(91.5)	38(84.4)	.263	48(78.7)	18(85.7)	.483
Searching for dietary treatment	87(72.5)	40(60.6)	.095	24(78.0)	29(64.4)	.128	41(67.2)	11(52.4)	.224
Changes in amount of intake									
• No change	47(39.2)	41(62.1)	.005**	25(42.4)	29(64.4)	.039*	22(36.1)	12(57.1)	.202
• Increase	18(15.0)	3(4.5)		10(16.9)	2(4.4)		8(13.1)	1(4.8)	
• Decrease	55(45.8)	22(33.3)		24(40.7)	14(31.1)		31(50.8)	8(38.1)	
Weight change									
• Gained	27(22.5)^5)^	10(15.2)	.073	16(27.1)	3(6.7)	.026*	11(18.0)	7(33.3)	.024*
• Lost	28(23.3)	9(13.6)		7(11.6)	8(17.8)		21(34.4)	1(4.8)	
• Maintained	65(54.2)	47(71.2)		36(61.0)	34(75.6)		29(47.5)	13(61.9)	

^a^ Extend cancer survivor = between two and five years after initial diagnosis; Long term cancer survivor = over five years after initial diagnosis

^b^ Kinds of eating problem include dysphasia, anorexia, dyspepsia, vomiting/nausea, constipation, xerostomia, hypogeusia, odor, pain and others.

^c^ Values are yes/ no

^d^ All values are n (%)

^e^ Significant difference at * (p<0.05) and ** (p<0.01) was found by Chi-square test for categorical variables.

### 3. Changes in health related factors

**Fig 1:**
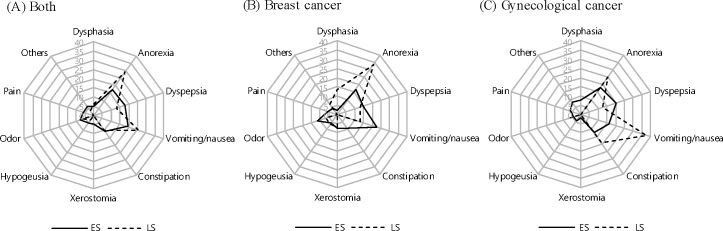
Types of eating problem among survivors by the survival stages among Korean female cancer patients.

**Fig 2:**
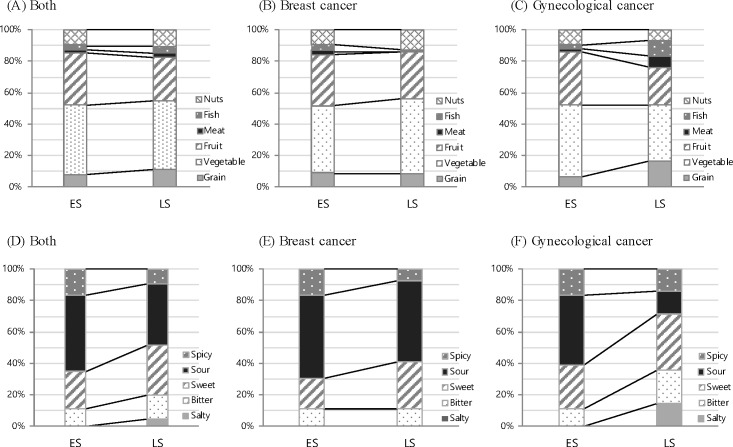
Types of food and taste preference by the survival stages among Korean female cancer patients.

Changes in health related factors of survivors in the recent 6 months are summarized in Table 2. ES cancer survivors reported significantly higher rates of eating problems, changes in intake, and changes in taste after medical treatment in the recent 6 months (p < .05). Furthermore, body weight had also significantly changed for recent 6 months in both breast and gynecologic cancer survivors: In the ES group, 27.1% of breast cancer survivors experienced an increase in body weight, whereas 34.4% of gynecologic cancer survivors showed a decrease (p < .05). Among breast cancer survivors, approximately 50% of the ES group and 25% of the LS group reported eating problems, and the main eating problems were vomiting/nausea in ES and anorexia in LS. In survivors of gynecologic cancer, the main problems were dyspepsia in the ES group and vomiting/nausea in the LS group (Fig 1). Among breast cancer survivors, the ES group reported high rates of changes in taste, food preference, and amount of intake (amount of food intake was decreased in 40% of ES breast cancer survivors) compared with the LS group (p < .05). Moreover, most of the survivors preferred fruits and vegetables at 77% in ES and 70.8% in LS, compared with other food groups (fish and meat intake was 5.5% in ES and 7.1% in LS) (Fig 2). Particularly, for breast cancer, the ES group had a greater preference for protein foods (fish and meat) than that of the LS group. In contrast, for gynecologic cancer, the LS group had a greater preference for protein food than the ES group. The ES group preferred the taste of sour, whereas the LS group preferred sweet tastes; this difference between LS and ES groups was greater for gynecologic cancer survivors (Fig 2).

### 4. Nutritional Care Needs

**Fig 3:**
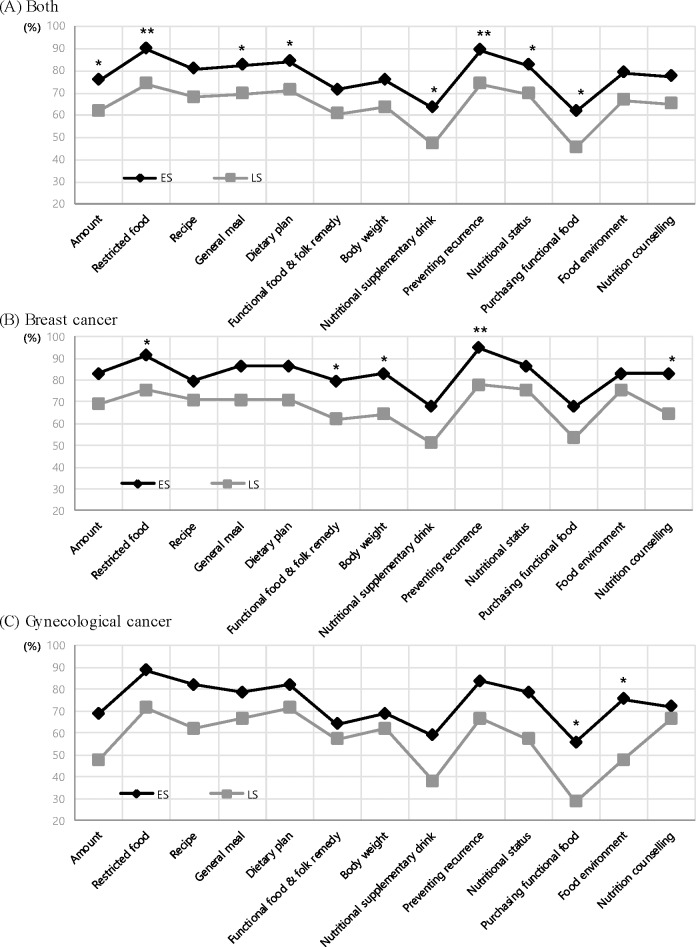
Nutritional care needs by the survival stages among Korean female cancer patients.

Approximately 90% of survivors demanded nutritional care and education in all groups (data not shown). Fig 3 shows typology of nutritional information needs containing fifteen categories of nutritional education. Various needs showed a significant difference between ES and LS. In the ES group of total cancer survivors, the most important nutritional needs were allowed and restricted foods (90%) and dietary patterns to prevent recurrence (89.2%,) (p < .01). The total ES group also reported significantly higher nutritional needs than LS in the following categories: amount of daily meals, usefulness for general meal, dietary plan, nutritional supplementary drink, information on improvement of nutritional status, and purchasing of health-functional food (p < .05). Among breast cancer survivors, the following five nutritional needs categories were significantly higher in ES compared with LS: allowed and restricted foods, information on functional foods, diet to control body weight, dietary pattern to prevent recurrence, and follow-up nutritional counseling (p < .05). In gynecologic cancer survivors, two nutritional needs categories; purchasing of functional food and information on improvement of food environment were significantly higher in ES compared with LS (p < .05).

### 5. Quality of Life

According to survival stage, long-term survivors among both cancer types especially, breast cancer survivors had a significantly higher SF-36 score (p < .01). The scores of the LS group were higher than those of the ES group, and the difference was greater for physical health than mental health. And then according to eating problem, the subjects who have eating problem had markedly lower quality of life in almost part (Table 3).

**Table 3:** Quality of life (SF-36)^a^ score by eating problem and by survival stage among Korean female cancer patients.

			Physical health	Mental health	Health-related QOL
Eating problem	Both	Having eating problem (n = 70)	51.7±25.4^b^	60.1±18.3	53.7±18.5
		Don’t having problem (n = 116)	62.5±23.3	69.3±16.9	67.5±17.9
		*p*-value	.005*^c^	.001*	.000*
	Breast cancer	Having eating problem (n = 38)	56.2±25.3	60.6±20.9	57.8±17.7
		Don’t having problem (n = 66)	61.5±23.9	69.6±16.7	69.0±17.5
		*p*-value	.297	.027*	.003*
	Gynecological caner	Having eating problem (n = 32)	46.4±24.9	59.5±15.1	48.4±18.4
		Don’t having problem (n = 50)	63.8±22.7	69.0±17.4	65.6±18.5
		*p*-value	.002*	.011*	.000*
Survival stage	Both	Extend stage (n = 120)	56.4±24.63	64.9±17.7	59.0±19.3
		Long-term stage (n = 66)	63.0±23.3	68.5±16.7	68.3±18.0
		*p*-value	.072	.169	.001*
	Breast cancer	Extend stage (n = 59)	57.6±24.0	64.1±19.6	60.6±17.9
		Long-term stage (n = 45)	62.2±24.9	69.2±17.3	70.5±17.5
		*p*-value	.337	.171	.006*
	Gynecological caner	Extend stage (n = 61)	54.3±26.1	64.6±17.6	57.5±20.6
		Long-term stage (n = 21)	64.7±20.0	67.1±15.6	63.4±18.5
		*p*-value	.167	.636	.293

^a^ SF-36: The 36-Item Short Form Survey has 8 categories, then they are divided again physical Component Summary(Physical functioning, Role physical, Bodily pain and General health) and Mental Component Summary(Vitality, Social functioning, Role emotional and Mental health). Each category score range from 0 to 100, and greater number indicating a better quality of life.

^b^ All values are mean±SD

^c^ Significant difference at * (p<0.05) was found by Chi-square test for categorical variables

## Discussion

This study sought to determine health related factors and nutritional care needs according to the stage of cancer survivorship and cancer type among female cancer survivors who complete medical treatments in South Korea. Cancer survivors have different aspects in various points including physical and psychological factors according to their survival stage of time elapsed [3, 7, 15]. Acute and extended stage survivors are interested in the side effects care, preventing recurrence, and healthy life, by comparison with long-term stage survivors’ main concerns are social and metal care [7]. Furthermore long-term stage survivors have significantly better quality of life than extended stage [15].

The diagnosis of cancer can markedly change an individual’s life. Cancer does not necessarily mean death with continued scientific progress and developments in health care [6]. Cancer survivors are highly motivated to improve their lifestyle and most dietary changes are healthful (e.g., eating more fruits and vegetables, and reducing the intake of fat) [18, 19]. In our study, nearly two-third of survivors had healthy eating behaviors with three regular meals and snaking once a day. As previous studies reported that the most common dietary changes among cancer survivors is eating more fruits and vegetables [19–21], these food groups were the most preferred food in our subjects. Over half of our survivors choose fruits and vegetables for their snack, and survivors in the ES had a strong preference for these foods rather than those of LS survivors. The cancer survivors did not have balanced nutrition; they ate immoderate amounts of fruits and vegetables compared with other food groups such as grains, meat, and fish. The result could be related with the general information for cancer patients and survivors, via media, internet, book, and others, because almost nutritional information includes “eating a lot of vegetables and fruits”. This unbalanced food intakes might impede recovery and negatively affect their health.

In terms of the stages of cancer survivorship, ES survivors had more diet-related problems than LS survivors; mainly, ES group reported dyspepsia and LS group had anorexia and vomiting/nausea, in addition, symptoms were specific in LS. The amount of food intake was decreased for various eating-related reasons, and these women exhibited greater taste preference changes. ES survivors preferred the taste of sour, whereas LS survivors preferred the taste of sweet and salty in gynecological cancer survivors. This finding also corresponds with the research of Rock et al.; the cancer patients who were completed medical treatments need nutritional care of anorexia, taste change and dyspepsia [22]. As the study of Lim and Han [16], low quality of life in ES survivors might be related to their diet-related problems. Moreover, the research to determine the relationship between doing nutritional guideline of American Cancer Society (ACS) and quality of life with SF-36 on cancer survivors reported the survivors who perform nutritional guideline were shown high rates of quality of life [23]. Also, all of the groups had high rates of nutritional care needs particularly in the categories of restricted food, preventing recurrence, nutritional status, and dietary plan. In addition, preventing recurrence is one of main concerns of cancer patients and survivors [7, 16, 22, 24].

Some variables had different aspects according to the survivorship stage and type of diagnosis. There were high rates of changes in food preference after medical treatment in the ES group of breast cancer survivors and the LS group of gynecologic cancer survivors. LS breast cancer patients showed an increased preference for vegetables, whereas those with gynecologic cancer showed an increased preference for fish, meat and grain. The variable of body weight change also differed according to the stage and diagnosis of cancer in both groups. In breast cancer survivors, over a quarter of the ES group reported body weight gain, and one of their main concerns and nutritional care demands was control of body weight. In contrast, over a third of the ES gynecologic cancer survivors reported weight loss.

According to previous studies, body weight gain is a common problem in breast cancer patients and survivors that can be associated with the kind of therapy, period of treatment, and life cycle: a number of patients and survivors receive chemotherapy as a part of their treatment, receive a prolonged course of high-dose medical treatment, and are premenopausal at diagnosis [7, 19]. However, in this study approximately half of the ES group with breast cancer reported a decrease in the amount of food intake after medical treatment because of anorexia and concerns over weight control. Song et al. reported that female survivors’ body weight control is relative with the symptoms of constipation [25].

Also our breast cancer survivors were motivated regarding health care and had a high rate of demand for nutritional needs compared with gynecologic cancer survivors. These findings are consistent with reported research, in which up to 98% of breast cancer survivors had experiences of positive lifestyle changes post diagnosis [24] and post-traumatic growth was significantly higher than in the healthy control group [26].

In addition, there are some national health/medical care systems for cancer patients in Korea with national health insurance systems as “Caner Control Act”, “Policy on Home-Based Management for Cancer” and “Policy on Hospice and Palliative Care for Terminal Cancer Patients” [27–29]. However cancer patients still cannot be given better service. Those medical care systems are graded payment, and insufficient to cover the all patients and survivors. Regarding diet and nutrition managements, several hospitals are carrying out nutritional care, counselling, and education program, but it is not responsibility. Even there are no statistical figures to investigate the supports’ implementation status.

A major strength of this study is the analysis of Korean female cancer survivors by survivorship stage for nutritional care after medical treatments. We focus on cancer survivors’ health related factors, including eating problems and nutritional care needs. Our study provides meaningful results for recent eating habits, changes in health related factors after surgery and medical treatments, and specific content of nutritional needs. Few studies have been conducted to find out the nutritional demands of Korean female cancer survivors, and even less information related to their survival stage. Therefore, studies performed after provision of nutrition care to meet the patients’ demands are needed to investigate the beneficial effects. We also added stratified analysis for correction, and there is no significantly difference in -health related factors by menopausal status and mean age.

Despite this strength, there are some limitations of this study. This is a cross-sectional design, which precludes inferences as to causation. The multiple-choice questionnaires are not able to define significant difference. In addition, we did not take into consideration the duration of eating problems, the type of medical treatments, and family history of cancer.

In conclusion, the cancer survivors had various diet-related problems by survival stage and type of cancer as dyspepsia in ES on both types of cancer, anorexia in LS on breast cancer and vomiting in LS on gynecological cancer. ES survivors had more changes in taste and their food consumption have decreased, and lower quality of life than those of LS. Survivors of both types and survival stage of cancer have changed body weight. Most of the survivors needed proper nutritional care particularly focused on the categories of restricted food and preventing recurrence. Therefore eating problem, body weight, nutritional demand and quality of life according to the survival stage and types of cancer should considered to the nutritional care and education for survivors’ health.

## Supporting Information

S1 Table Cancer stage and medical history according to survival stage among Korean female cancer patients. (XLSX)

S2 Table Eating habits according to survival stage among Korean female cancer patients. (XLSX)

S3 Table Changes in dietary factors after surgery and medical treatments by the years Korean female cancer patients. (XLSX)

S4 Table Changes in food and taste preference. (XLSX)

S5 Table The necessity of nutrition education. (XLSX)

S6 Table Important contents for nutrition management. (XLSX)

S7 Table Healthy-related QOL(SF-36)1) score by eating problem and by survival stage among Korean female cancer patients. (XLSX)

S8 Table Changes in dietary factors after surgery and medical treatments by the years Korean female cancer patients. (XLSX)
